# ACE Inhibitors Potently Reduce Vascular Inflammation, Results of an Open Proof-Of-Concept Study in the Abdominal Aortic Aneurysm

**DOI:** 10.1371/journal.pone.0111952

**Published:** 2014-12-04

**Authors:** Kim E. Kortekaas, C. Arnoud Meijer, Jan Willem Hinnen, Ronald L. Dalman, Baohui Xu, Jaap F. Hamming, Jan H. Lindeman

**Affiliations:** 1 Department of Vascular Surgery, Leiden University Medical Center, Leiden, The Netherlands; 2 Division of Vascular Surgery, Stanford Medical School, Stanford, California, United States of America; 3 Department of Vascular Surgery, Jeroen Bosch Ziekenhuis, ‘s Hertogenbosch, The Netherlands; University of Milan, Italy

## Abstract

**Background:**

Independent of their blood pressure lowering effect, ACE inhibitors are thought to reduce vascular inflammation. The clinical relevance of this effect is unclear with the current knowledge. Abdominal aortic aneurysms (AAA) are characterized by a broad, non-specific inflammatory response, and thus provide a clinical platform to evaluate the anti-inflammatory potential of ACE inhibitors.

**Methods and Results:**

Eleven patients scheduled for open AAA repair received ramipril (5 mg/day) during 2–4 weeks preceding surgery. Aortic wall samples were collected during surgery, and compared to matched samples obtained from a biobank. An anti-inflammatory potential was evaluated in a comprehensive analysis that included immunohistochemistry, mRNA and protein analysis. A putative effect of ACE inhibitors on AAA growth was tested separately by comparing 18-month growth rate of patients on ACE inhibitors (n = 82) and those not taking ACE inhibitors (n = 204). Ramipril reduces mRNA expression of multiple pro-inflammatory cytokines such as IL-1β, IL-6, IL-8, TNF -α, Interferon-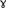, and MCP-1, as well as aortic wall IL-8 and MCP-1 (P = 0.017 and 0.008, respectively) protein content. The is followed by clear effects on cell activation that included a shift towards anti-inflammatory macrophage (M2) subtype. Evaluation of data from the PHAST cohort did not indicate an effect of ACE inhibitors on 18-month aneurysm progression (mean difference at 18 months: −0.24 mm (95% CI: −0.90–0.45, P = NS).

**Conclusions:**

ACE inhibition quenches multiple aspects of vascular inflammation in AAA. However, this does not translate into reduced aneurysm growth.

**Trial Registration:**

Nederlands Trial Register 1345.

## Introduction

Independent of their blood pressure lowering effects, ACE inhibitors are thought to reduce vascular inflammation. [1]–[4] It has been suggested that this off-target anti- inflammatory (pleiotropic) effect contributes to the efficacy of this class of anti-hypertensives. Although an anti-inflammatory potential of ACE inhibitors has been firmly established in *in vitro* studies, [5]–[7] it is still unclear whether and how these observations translate to the human situation. [8] The abdominal aortic aneurysm (AAA) is part of the atherosclerotic spectrum of diseases. The pathology is characterized by a comprehensive, localized inflammatory response that is held responsible for the progression and complications of the disease. [9], [10] Unlike the occlusive forms of atherosclerotic disease, hypertension is very weakly associated with incident AAA disease [11] whereas AAA progression is not hypertension related. [12] As such the condition provides an opportunity to test the anti-inflammatory potential of ACE inhibitors independently from an effect on blood pressure.

Animal studies show that ACE-inhibitors effectively quench aortic inflammation and prevent aneurysm formation in *rodent* models of AAA disease. [5]–[7], [13] Human data on the other hand is less clear. A retrospective case-control study using a large Canadian administrative database showed that patients with AAA treated with ACE inhibitors, but not those treated with other anti-hypertensives are less likely to present with ruptured AAA. [3] In contrast, a study by Wilmink failed to observe a beneficial effect of ACE inhibitors on aneurysm progression, [15] whereas Sweeting et al. [16] observed accelerated aneurysm growth in patients taking ACE inhibitors.

Because of this controversy, and the absence of molecular data for the human situation, we considered an evaluation of the anti-inflammatory potency of ACE inhibitors relevant. To that end, we first studied the anti-inflammatory potential of regular dose ACE inhibition through ramipril in the context of AAA. A possible effect of ACE inhibitors on AAA growth was evaluated in a sub analysis of the data available from PHAST; a trial evaluating the effect of doxycycline on AAA progression. [17] Results of these studies show that ACE inhibitors have profound anti-inflammatory effects on aspects of vascular inflammation, resulting in reduced expression of pro inflammatory cytokines and attenuated cell activation (in particular macrophages). However, these anti-inflammatory effects are not followed by an effect on AAA growth.

## Materials and Methods

### Patient populations

This open proof-of-concept study was approved by the Medical Ethical Committee of the Leiden University Medical Center. Written informed consent was obtained from all patients. Patients scheduled for open AAA repair and not taking ACE inhibitors or AT II antagonists were eligible for the study. Decision for open-repair was based on anatomical (e.g. neck, elongation), and patients characteristics (e.g. age) and preferences. Patients with hypotension (diastolic blood pressure <80 mm Hg), kidney dysfunction (estimated clearance <30 mL/min), chronic inflammatory disease or (suspected) so-called inflammatory aortic aneurysms, were excluded from participation in the study. The study was started in January 2008 and the final patient was included in September 2009. This study was not registered as proof-of-concept study is not considered a clinical trial by the Dutch regulatory authorities.

Patients received ramipril 5 mg once a day in the 2–4 weeks preceding their planned elective open repair. The final dose was taken in the evening before the surgery. Control AAA wall samples were obtained from the LUMC biobank, these samples were matched with the ramipril group for sex, age, maximum AAA diameter and statin use. None of the patients in the control group was using ACE inhibitors or Angiotensin II antagonists. AAA wall tissue was taken from the anterior-lateral aneurysm wall at the level of the maximal diameter of the aneurysm. All wall samples (i.e samples both study samples and biobank samples) were collected immediately after opening of the aneurysm sac. Adhering thrombus was carefully removed and wall samples were immediately halved. One half was snap-frozen in CO2-cooled iso-pentane or liquid nitrogen and stored at −80°C until use for mRNA (RT-PCR) and protein (ELISA) analysis. The other half was fixed in formaldehyde (24 hours), decalcified (Kristensens solution, 120 h), and paraffin embedded for histological analysis. [10] All analyses were performed in an investigator-blind fashion.

The effect of ACE inhibition on aneurysm progression was evaluated separately in patients participating in the Pharmaceutical Aneurysm Stabilization Trial (PHAST) (registered in the Nederlands trial registry: http://www.trialregister.nl/trialreg/admin/rctview.asp?TC=1345). PHAST is a placebo controlled multi-center trial of 18 months doxycycline vs. placebo in patients under surveillance for a small AAA (35–50 mm) with aneurysm progression as primary endpoint. [17] The CONSORT diagram for this study is available as supporting information; see Figure S1. Primary approval for the study was granted by the Medical and Ethical Committee of the Leiden University Medical Center, The Netherlands. All local review boards of the participating centers approved the study. Written informed consent was obtained from all participants. Inclusion of patients commenced in Oktober 2008 and the study was completed in June 2011. A consort diagram of the study is provided in reference 17. PHAST included 286 patients. Eighty two of these patients were on ACE inhibitors and 204 were not using an ACE inhibitor. AAA progression was measured by standardized ultrasound at 6-month intervals over an 18 month period. In order to avoid intra-observed variation, all measurements were made by a single observer. Decisions for ACE inhibitors were based on patient characteristics, local policies, and patient preferences and were not influenced by participation in the study. A consort diagram for PHAST is provide in reference 17.

### Immunohistochemistry

Slides were incubated overnight with antibodies against myeloperoxidase (MPO; rabbit polyclonal, 1∶4000 dilution, DakoCytomation, Heverlee, Belgium), CD3 (polyclonal rabbit, 1∶400 dilution, Abcam, Cambridge, UK), CD4 (clone 4B12, 1∶200 dilution, DakoCytomation), CD8 (clone C8/144B, 1∶200 dilution, DakoCytomation), CD20 (clone L26, 1∶1000 dilution, DakoCytomation), CD68 (clone KP6, 1∶1200, DakoCytomation), and CD138 (clone B-B4, 1∶1000 dilution, Serotec, Oxford, UK). [10] Macrophage activation was assessed by double staining. CD68 (clone L26, DakoCytomation)/iNOS (inducible nitric oxide synthase; rabbit polyclonal AB3523, 1∶400 dilution, Abcam Cambridge, UK) double staining was used to characterize M1 macrophages,7 and CD68/CD163 ((Clone 10D6, 1∶400 dilution, DakoCytomation) staining was used to specify M2 macrophages. Double staining for CD68/HLA-DR (human leukocyte antigen DR; clone TAL.1B5, 1∶1000 dilution, DakoCytomation) was performed in order to assess macrophage activation. [18]–[21] The double-stainings were quantified by counting the number of double positive cells in six representative medium power fields (two photographs for each vascular layer) by two independent blinded observers. There was high inter observer reliability for the CD68-CD163, CD68-iNOS, and CD68-HLADR (*α* = 0.916, *α* = 0.875, *α* = 0.839, respectively). Relative changes in immunoreactivity of the mono-stainings were quantified using ImageJ software (version 1.37c, NIH, USA). Six representative medium power fields (two photographs for each vascular layer) were photographed at a 20x magnifier. Thereafter, images were subjected to color deconvolution to exclude background noise from analysis. The relative area of the specific staining to the total area was determined by using threshold segmentation. The threshold was separately determined for each staining, to exclude background immunoreactivity. Representative images for the double staining are shown in Figure S2. Absolute cell contents are expressed as number of cells per mm^2^.

### Semi quantitative mRNA analysis

Total RNA extraction was performed using RNAzol (Campro Scientific, Veenendaal, The Netherlands) and glass beads. [22] Copy-DNA was prepared using kit #A3500 (Promega, Leiden, The Netherlands) and quantitative real-time polymerase chain reaction (Taqman system) analysis was performed for human interleukin (IL)-1α, IL-1β, IL-6, IL-8, IL18 Tumor necrosis factor (TNF) -α, Interferon-, MCP-1, perforin, granzyme A, BLIMP-1, MAD4, immunoglobulin linkerprotein, MMP (matrix metalloproteinase)-9 and 12, the Cathepsins K, L and S, the collagens type I and III, Plasminogen Activator Inhibitor I (PAI-1) and the angiotensin receptor I on the ABI-7500 Fast system (Life Biosciences, Nieuwerkerk aan den IJssel, The Netherlands) using established primer/probe sets (Assays on Demand, Life Biosciences) and Taqman Gene Expression Master Mix (Life Biosciences).

Analyses were performed according to the manufacturer’s instructions. Glyceraldehyde-3-phosphate dehydrogenase (GAPDH) expression was used as a reference and for normalization [23].

### Protein analysis

Aortic wall tissues were pulverized in liquid nitrogen and homogenized in two volumes lysis buffer (10 mM Tris pH 7.0, 0.1 mM CaCl2, 0.1 M NaCl, 0.25% (v/v) Triton X-100). Samples were subsequently centrifuged at 10,000 *g* for 15 minutes at 4°C, and the supernatant protein extract was snap-frozen in liquid nitrogen and stored at –80°C until analysis. Protein content in thawed protein extracts homogenates was determined with a BCA protein assay kit (Pierce, Rockford, IL, USA). Cytokine/chemokine protein levels in these homogenates were measured by separate ELISAs for IL-6, IL-8 (PeliKane compact kit, Sanquin, Amsterdam, The Netherlands), and MCP-1 (Quantikine kit, R&D Systems, Abingdon, UK).

### Statistical analysis

Statistical analyses were performed with SPSS19.0 (SPSS Inc., Chicago, IL, USA). For the comparisons, a P-value <0.05 was considered statistically significant. Differences between the groups were evaluated by ANOVA (normally distributed data) or the Kruskal Wallis test in case of non-normally distributed continuous data. The Benjamini-Hochberg correction was applied on the ELISA, PCR, and immunohistochemistry data to correct for multiple comparisons.

The effects of ramipril therapy in the PHAST trial were analysed using linear mixed model analyses to examine a possible effect of ramipril therapy on changes in aneurysm diameter during the follow-up period. [17], [24] Random terms in the models were patient, follow up time and the square of follow-up time, with an unstructured covariance matrix. Fixed terms were follow up, follow up squared, and the interactions of the latter two with treatment group. Baseline diameter was used as dependent variable. The model did not allow a systematic difference at baseline between the treatment groups. The effect of ramipril treatment on aneurysm growth at 18 months follow-up, was estimated and tested based on this model, as was the difference at 6 and 12 months. Models were validated both graphically by residuals analysis and analytically by extending the models with more terms.

## Results

### Patient population

Baseline characteristic of the pre-operative ramipril-intervention group (n = 11) and the matched control group (n = 11) used in the molecular analysis are shown in table 1.

**Table 1:** Baseline patient characteristics of the Ramipril intervention study.

	Ramipril	Controls
	N = 11	N = 11
Age (year)	67 [64–77]	71 [64–77]
AAA diameter (mm)	57 [51–67]	60 [56–71]
Male sex	9/10	9/10
Statin use	6/10	6/10

Median [inter quartile range].

A possible effect of ACE-inhibitors was evaluated separately in patients participating in the Pharmaceutical Aneurysm Stabilization Trial (PHAST). [17] Baseline characteristics of these participants are shown in table 2. The cohort includes 82 patients who were using an ACE inhibitor and 204 patients not using ACE-inhibitors. Patients using an ACE inhibitor and those not using an ACE inhibitor had similar baseline characteristics with respect to gender, age, smoking status, and AAA diameter, but were dissimilar with respect to systolic blood pressure and diabetic disease (Table 2).

**Table 2:** Baseline patient characteristics of the PHAST population.

	Ace inhibitor	Controls
	N = 82	N = 204
Age (yr)	70 [66–75]	71 [65–75]
AAA diameter (mm)	43.0 [39.0–48.0]	43.0 [39.0–47.0]
Male sex	74 (90.2%)	177 (86.8%)
Smoking (current/never/former) %	30.5/8.5/61.0	36.8/10.8/52.5
BMI (kg/m2)	26.3 [24.3–28.5]	27.0 [24.6–28.7]
ApoA (g/L)	1.3 [1.2–1.5]	1.4 [1.3–1.6]
ApoB (g/L)	0.9 [0.8–1.0]	0.9 [0.8–1.1]
Creatinine (µmol/L)	92.0 [78.5–109.0]	89.0 [75.5–103.0]
Triglycerides (mmol/L)	1.9 [1.4–2.7]	1.8 [1.4–2.5]
Cholesterol (mmol/L)	4.6 [4.0–5.6]	4.7 [4.1–5.4]
Diabetes*)	18.3%	13.2%
Diastolic blood pressure (mm/Hg)	84.5 [78.0–90.0]	85.0 [80.0–90.0]
Systolic blood pressure (mm/Hg) *)	143.0 [132.0–159.5]	150.0 [130.0–170.0]

Median [inter quartile range].

*) significant difference between the two groups (P<0.05). Abbreviations: BMI, body mass index; ApoA, apolipoprotein A; ApoB, apolipoprotein B.

### Pre-operative ramipril therapy and the inflammatory response in AAA

**Figure 1:**
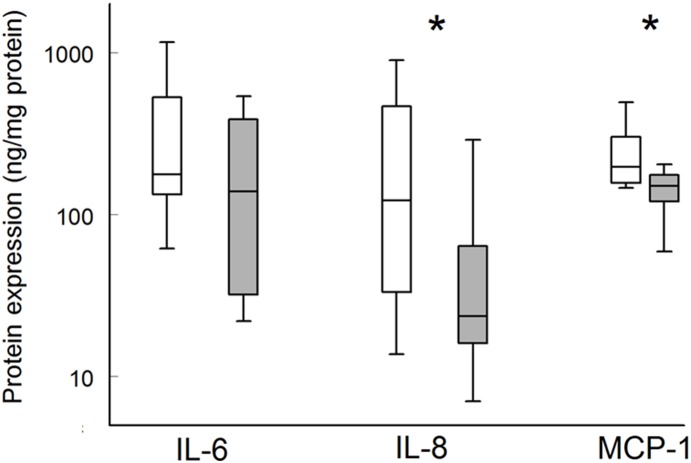
Aneurysm wall protein interleukin-6, interleukin-8, and monocyte chemoattractant protein 1 content. (*) levels significantly lower in Ramipril-treated individuals (P<0.014 and P<0.008 for IL-8 and MCP-1 respectively). Non-treated controls (white bars); Ramipril-treated patients (grey bars).

Ramipril treatment did not influence the expression of the AT1 receptor (log transcript level relative to GAPDH (GAPDH = 0): −2.38 [−2.94– −1.94] and −2.06 [−2.70– −1.04] (median [inter quartile range]) in the ramipril and control group respectively). Treatment was associated with a comprehensive suppression of mRNA expression of pro-inflammatory cytokines as well as of read-outs of TGFβ signalling (Table 3), yet the latter effect was lost upon correction for multiple testing. Protein analysis (ELISA) for IL-6, -8 and MCP-1 showed that this suppression is paralleled by reduced protein levels (P<0.014 and P<0.008 for IL-8 and MCP-1 respectively, Figure 1). The apparent reduction in IL-6 did not reach statistical significance (Figure 1, P = 0.16).

**Table 3:** Relative mRNA expression of selected inflammatory mediators, proteases, cytokines, and cell activation markers (log transcript level relative to GAPDH (GAPDH = 0)).

		Ramipril treatment	Controls	P-value
		N = 10* ^)^	N = 11	
	IL-1α	–2.68 [–3.02– −2.37]	–1.60 [–2.03– −1.26]	0,00003* ^)^
	IL-1β	–1.28 [–1.44– –1.15]	–0.73 [–1.01– −0.45]	0,00002* ^)^
	IL-6	–0.97 [–1.35–0.56]	–0.33 [–0.59–0.08]	0,002* ^)^
Cytokines	IL-8	–1.06 [–1.43– −0.77]	–0.17 [–0.43–0.13]	0,00002* ^)^
	IL-18	–1.05 [–1.42– −0.87	0.84 [–1.03–0.50]	0.095
	TNF α	–1.92 [–2.18– −1.77]	–1.46 [–1.75– −1.05]	0,002* ^)^
	MCP-1	–0.74 [–0.99– −0.31]	0.11 [–0.41–0.75]	0.001* ^)^
	IFN γ	–2.34 [–2.53– −2.12]	–1.43 [–1.94– −0.98]	0.001* ^)^
Cytotoxic T cell	Perforin	–1.93 [–2.02– −1.85]	–1.31 [–1.47– −0.93]	0.002* ^)^
	Granzyme A	–1.69 [–1.90– −1.56]	–0.87 [–1.26– −0.18]	0,001* ^)^
	MAD-4	–1.13 [–1.20– −1.10]	–0.82 [–0.92– −0.51	0.008* ^)^
B−/Plasma cell	BLIMP-1	–0.56 [–0.70– −0.43]	0.00 [–0.37–0.40]	0.02
	IgG linker protein	1.32 [1.02–1.75]	2.08 [1.75–2.40]	0.002* ^)^
Macrophage	MMP-12	–1.27 [–1.82– −0.82]	–0.76 [–1.36– –0.46]	0.19
	Cathepsin K	–0.98 [–1.17– –0.76]	–0.77 [–1.00– −0.64]	0.12
	MMP-2	0.01 [–0.25–0.18]	0.07 [–0.15–0.42]	0.86
	MMP-3	–1.68 [–2.32– −1.25]	–0.98 [–1.77– −0.25]	0.03
Proteases	MMP-9	–0.45 [–0.65– −0.28]	0.02 [–0.37–0.48]	0.009* ^)^
	Cathepsin S	–0.82 [–0.88– −0.66]	–0.12 [–0.58–0.07]	0.001* ^)^
	Cathepsin L	–0.54 [–0.70– −0.36]	0.31 [–0.21–0.54]	0.0003* ^)^
TGF-β signaling	PAI-1	–0.54 [–0.93–0.02]	0.08 [–0.31–0.45]	0.031
	Collagen Type I	0.19 [–0.01–0.36]	0.72 [0.40–1.08]	0.034
	Collagen Type III	0.59 [0.27–0.92]	1.32 [0.93–1.69]	0.019

*^)^Significance reached after Benjamini-Hochberg correction.

Abbreviations: IL, interleukin; TNF-α, tumor necrosis factor-α; MCP-1, monocyte chemotactic protein-1; IFN-γ, interferon-γ; MMP, matrix metalloproteinase; TGF-β, transforming growth factor β; PAI-1, plasminogen activator inhibitor-1.

*^)^Snap frozen material was available from 10 patients.

**Figure 2:**
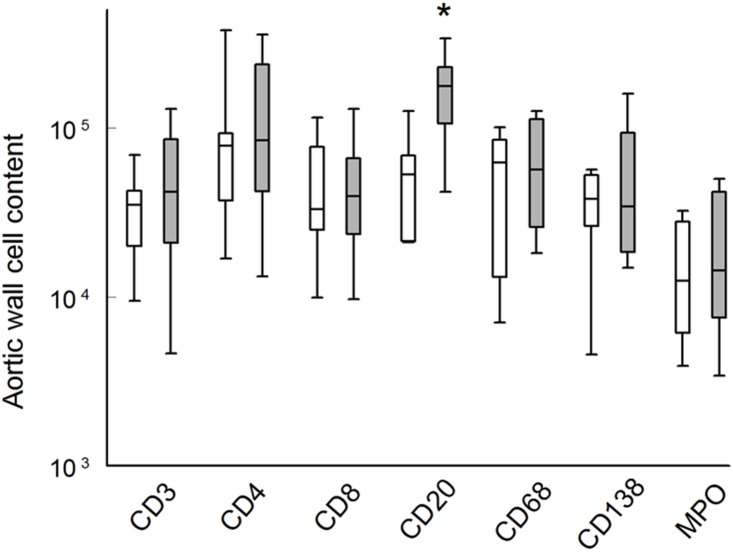
Effect of ramipril on aneurysm wall leucocyte content. Semi-quantitative analysis of aortic wall monocyte/macrophage (CD68), neutrophil (myeloperoxidase (MPO)), B-cell (CD20), plasma cell (CD138), T-cell (CD3), T-helper cell (CD4), and cytotoxic T-cells (CD8). Cell counts are based on reflect the number of double positive cells per 6 medium power fields. Cell content is expressed as the number of cells per mm^2^. Non-treated controls (white bars); ramipril-treated patients (grey bars). *P<0.009.

**Figure 3:**
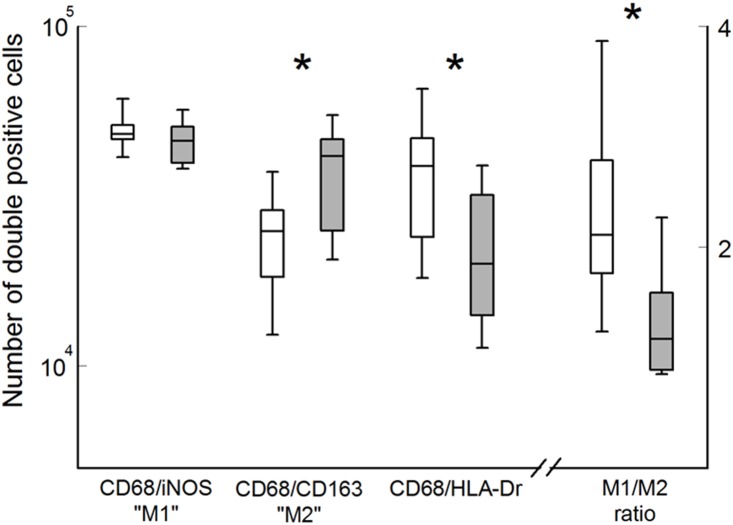
Ramipril reduces (P<0.004) macrophage activation as assessed by CD68/HLA-Dr double staining and increases aortic wall M2 content (CD68/CD163 double positive cells, P<0.006) content, thus resulting in a shift in the M1/M2 balance (P<0.002). Cell counts are based on the number of double positive cells per 6 medium power fields. Cell content is expressed as the number of cells per mm^2^. Non-treated controls (white bars); ramipril-treated patients (grey bars).

With the exception of a small increase in the aortic wall B-cell (CD20+) content, the reduction of cytokine levels was not paralleled by a quantitative effect on aortic wall inflammatory cell content. Viz., no difference was found in macrophage (CD68+), neutrophil (MPO+), T-cells (CD3+), T-helper cell (CD4+), cytotoxic T-cell (CD8+), and plasma cell (CD138+) content (Figure 2). Yet, ramipril treatment had a clear qualitative effects on the level of macrophage activation as illustrated by the shift in M1/M2 balance towards alternatively activated M2 macrophages (Figure 3), and reduced macrophage activation as assessed by CD68-HLA-Dr double staining (Figure 3), as well as reduced mRNA expression of macrophage activation markers such as MMP9, and the cathepsins L and S (Table 2). Along these lines we also observed quenching of the cytotoxic T cell activation markers perforin and granzyme A, and of the B-cell activation markers BLIMP-1, Mad-4, and Ig-linker protein.

### ACE-inhibition and aneurysm growth

**Figure 4:**
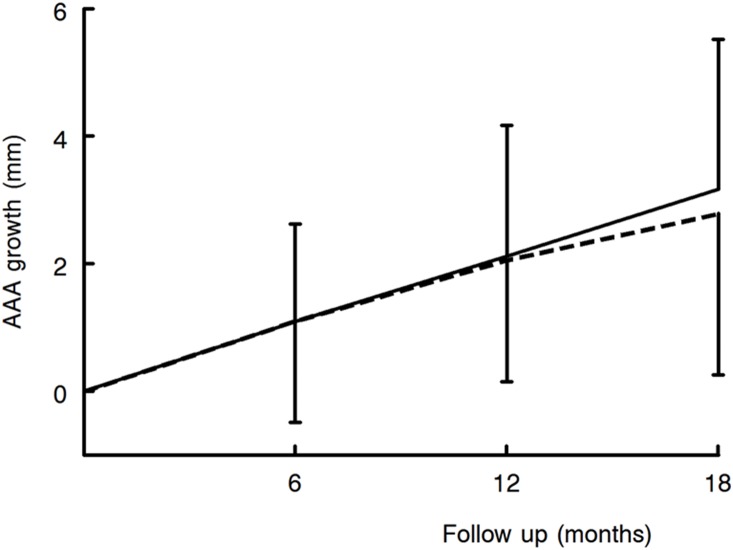
Similar aneurysm progression in AAA patients using an ACE inhibitor (dashed line, n = 82) and those not using an ACE-inhibitor (solid line, n = 204) (data from the PHAST study^17^). The mean 18 month difference between AAA patients on an ACE inhibitor and those not was −0.24 mm (95% CI: −0.90–0.45 mm).

We used the PHAST cohort to test whether the effects on aneurysm wall inflammation observed in the intervention study result in an effect on aneurysm progression. This analysis showed similar progression in patients treated with an ACE inhibitor and those not using an ACE inhibitor (difference at 18 months −0.24 mm (95% confidence interval: −0.90–0.45 mm, Figure 4). Because of the overrepresentation of patients with diabetic disease in the ACE-inhibitor group, and the apparently reduced AAA progression in patients with diabetic disease on aneurysm progression we repeated the analysis after exclusion of patients with diabetic disease. This did not influence the results of the analysis (results not shown).

## Discussion

This study determines that a brief episode of ACE inhibitor therapy through 5 mg/day ramipril exerts comprehensive anti-inflammatory effects on vessel wall inflammation in AAA disease. The effect is followed by a general reduction of cell activation. A possible effect of ACE-inhibitors on AAA growth was evaluated in a sub analysis of data from the PHAST trial, [17] a study aimed at evaluating the effect of doxycycline treatment in patients under surveillance for a small aneurysm. This evaluation did not indicate an effect of ACE inhibitors on aneurysm growth.

An abundance of preclinical evidence shows that the effects of ACE-inhibitors extend beyond blood pressure lowering. [1]–[4] It has been suggested that the clinical efficacy of these compounds is in part related to the so called pleiotropic anti-inflammatory effects. An effect that may in part relate to suppression of angiotensin II activity within the vessel wall, and that appears to involve quenching of NFκB mediated inflammation [2].

To the best of our knowledge all evidence is based on preclinical studies, and a relevant anti-inflammatory effect in the human context remains unclear with the current literature. 8 In this study we used the abdominal aortic aneurysm as a clinical model of vascular inflammation. This pathology is characterized by a comprehensive inflammatory response that involves multiple aspects of the inflammatory response. A proportion of AAA patients still undergoes planned, preventive open repair; thus providing a window for medical intervention. The validity of the concept has been previously proven for compounds such as statins and doxycycline. [21], [25] In this study we evaluated the potential of ramipril, one of more lipophilic members of the ACE-inhibitor family. This compound was chosen for its preclinical efficacy and its superior tissue penetration [26], [27].

Results of this proof-of-concept study show that a relatively brief period of 2–4 weeks ramipril treatment exerts potent anti-inflammatory effects on the aneurysm wall. A clear effect on both MCP-1 and IL-8 levels suggest that part of the effect is mediated by quenching of NFκB activity, thus following earlier preclinical observations showing that ramipril quenches NFκB activity in monocytes and endothelial cells. [28]–[32] The effect on inflammatory mediators was followed by clear effects on cell activation with a shift in macrophage signature towards dominance of alternatively activated (M2) macrophages. This shift may (partly) account for the reduced expression of the proteases MMP9, cathepsin L and S which are all considered instrumental in the process AAA growth. [33]–[37] An effect of ramipril on cellular activation was not restricted to macrophages as indicated by a broad reduction in the expression of markers of cytotoxic T-cell and B-cell activation, cell types that are also involved in the process of AAA formation and progression [9], [10].

A critical question is whether and how these clear effects translate into clinical effect such as growth and rupture in the context of AAA, and cardiovascular events in the context of atherosclerosis. There is a remarkable inconsistency with respect to a role of ACE inhibitors in aneurysmal disease. A survey in the administrative databases of Ontario suggests that users of ACE-inhibitors, but not of other classes of anti-hypertensives were less likely to present with a ruptured AAA (relative risk: 0.82, 95% CI 0.74–0.90). [14] Contrasting findings are reported by Sweeting et al. [16] who prospectively studied 1701 patients enrolled in the UK Small Aneurysm Trial. The authors concluded that mean aneurysm growth rate in the 169 patients taking ACE inhibitors at baseline was higher than that in the remaining controls (difference 0.63 mm/y, 95% CI 0.16–1.10 mm). [16] Wilmink et al. [15] followed 468 AAA patients identified in a screening program and observed no significant differences in growth rates in cases exposed to any of the main classes of antihypertensive drugs. Yet, the authors do report an increased collagen turnover in subjects receiving angiotensin-converting enzyme (ACE) inhibitors: 4.26 mg/L (95% CI, 3.73–4.79) as compared with 3.62 mg/L (95% CI, 3.49–3.76) for subjects not receiving ACE inhibitors. [15] Altogether, conclusions of these studies are contradictory and confusing. An effect of ACE inhibition (through perindopril) on AAA is currently under investigation in the Aardvark study http://clinicaltrials.gov/show/NCT01118520 which commenced in 2011–2012.

In anticipation of the results from this study we performed a sub-analysis in the PHAST cohort. PHAST is a prospective placebo-controlled study of 18-months doxycycline treatment on small aneurysm progression. [17] PHAST has the advantage of a meticulous follow up with minimal measurement variation (1.8 mm). A sub-analysis on patients who were on ACE inhibition for the total study period and those not on an ACE inhibitor revealed similar AAA progression in the two groups, thus challenging a major effect of ACE inhibition on aneurysm progression.

This conclusion follows the conclusion of Wilmink et al., [15] but is remarkable as the anti-inflammatory potency of ACE-inhibition through ramipril exceeds that of any of the other interventions reported to date, *and* as its activity also includes suppression of MMP9 and cathepsin S expression, proteases that are thought to be critically involved in the process of aortic wall debilitation [23], [33]–[37].

This apparent controversy may (in part) relate to the multiple facets of the angiotensin system. Angiotensin II plays a major role in matrix homeostasis through processes that include stimulation of TGFβ/SMAD signaling. [38] As such the ramipril intervention may result in reduced aneurysm wall TGFβ/SMAD signalling. Formally (viz. after correction for multiple testing) our study does not indicate an effect on the individual read-outs of TGFβ/SMAD signalling. Yet, this negative conclusion presumably reflects a major limitation of correcting for multiple comparisons in the context of related/explorative data, [39] as such we feel that the parallel reduction of all three markers of TGFβ/SMAD signalling reflects a genuine effect of ramipril. Consequently, the apparent failure of ACE-inhibition may result from counter balancing effects of matrix degradation (inflammation/proteases) and matrix deposition (TGFβ/SMAD signalling).

Yet, it is also important to note that the absence of an effect on AAA progression is in line with earlier observations from studies in patients receiving doxycycline, [17] a mast cell inhibitor (http://esvs.org/social/esvs-symposia-0), statin therapy, [21] or comprehensive immune suppression. [40], [41] These studies all show that interference with pro-inflammatory cascades does not result in attenuation of aneurysm growth, on the contrary we and others observed accelerated aneurysm growth in patients receiving doxycycline [17] or comprehensive immune suppression. [40] As a consequence these observations challenge the current concepts of aneurysm growth [41].

This interventional study has several limitations. The study is not placebo controlled and small. Yet given the progressive decline in open AAA repair procedures, interventional studies like this become more and more difficult to perform. We choose for a matching procedure for cases and control material from our tissue bank; this procedure reduced the clinical variation in the study and allowed for more firm conclusion. A larger sample size would have resulted in smaller confidence intervals and we cannot exclude that the ambivalent findings for IL-6 protein levels reflect lack of sufficient power. Another limitation is that we based the conclusion of the effects of ACE-inhibition on a sub analysis of the PHAST trial. This trail was not designed to test an effect of ACE inhibitors on aneurysm growth. Decisions for medical therapy in the trial were made by the attending physicians as result the group is heterogeneous with respect to the type of ACE-inhibitor and the dose. The two groups in the sub analysis were different with respect to systolic blood pressure. Yet as neither a meta analysis [12] nor unpublished data from the PHAST trial identify blood pressure as an determinant of aneurysm growth, it is unlikely that this will influence the results of the analysis. ACE inhibitors are a preferred class of antihypertensives in diabetic disease. As such the number of patients with diabetic disease was higher in the ACE inhibitor group. Diabetic disease is associated with reduced aneurysm progression, and consequently overrepresentation of diabetic patients may influence the conclusion of the sub analysis in the PHAST cohort. We therefore re-evaluated an effect of ACE-inhibitors after excluding the diabetic patients. This did not influence the conclusions of the sub analysis.

Conclusions of this study should be considered in the light of AAA disease. It cannot be ruled out that the observed pleiotropic anti-inflammatory effects are beneficial in the context of atherosclerosis. Yet, as a recent meta-analysis of trial data of anti-hypertensives failed to document class differences beyond that of blood pressure lowering [42] as such an effect in the context of atherosclerosis are either modest or limited to specific aspects of the disease.

## Supporting Information

Figure S1 **Consort diagram of the PHAST study** [17].
(TIF)

Figure S2 **Typical examples of the histological stainings.** A) isotype control CD68 antibody, B) CD 68 staining; C) isotype control CD163 antibody; D) CD163 staining; E) isotype control iNOS antibody; F) isotype control iNOS antibody; G) isotype control HLA-Dr antibody; H) HLA-Dr staining; I) CD68 (Red)/CD163 (Blue) double staining (arrows indicate double positive cells); J) CD68 (Red)/iNOS (Blue) double staining; K) CD68 (red)/HLA-DR (blue) double staining. Arrows in I, J and K indicate double positive cells.
(EPS)
