# Supplementary figures and images for: ACE Inhibitors Potently Reduce Vascular Inflammation, Results of an Open Proof-Of-Concept Study in the Abdominal Aortic Aneurysm
Source: PLoS One. 2014 Dec 4;9(12):e111952. doi: 10.1371/journal.pone.0111952 (PMC4256371; doi:10.1371/journal.pone.0111952)

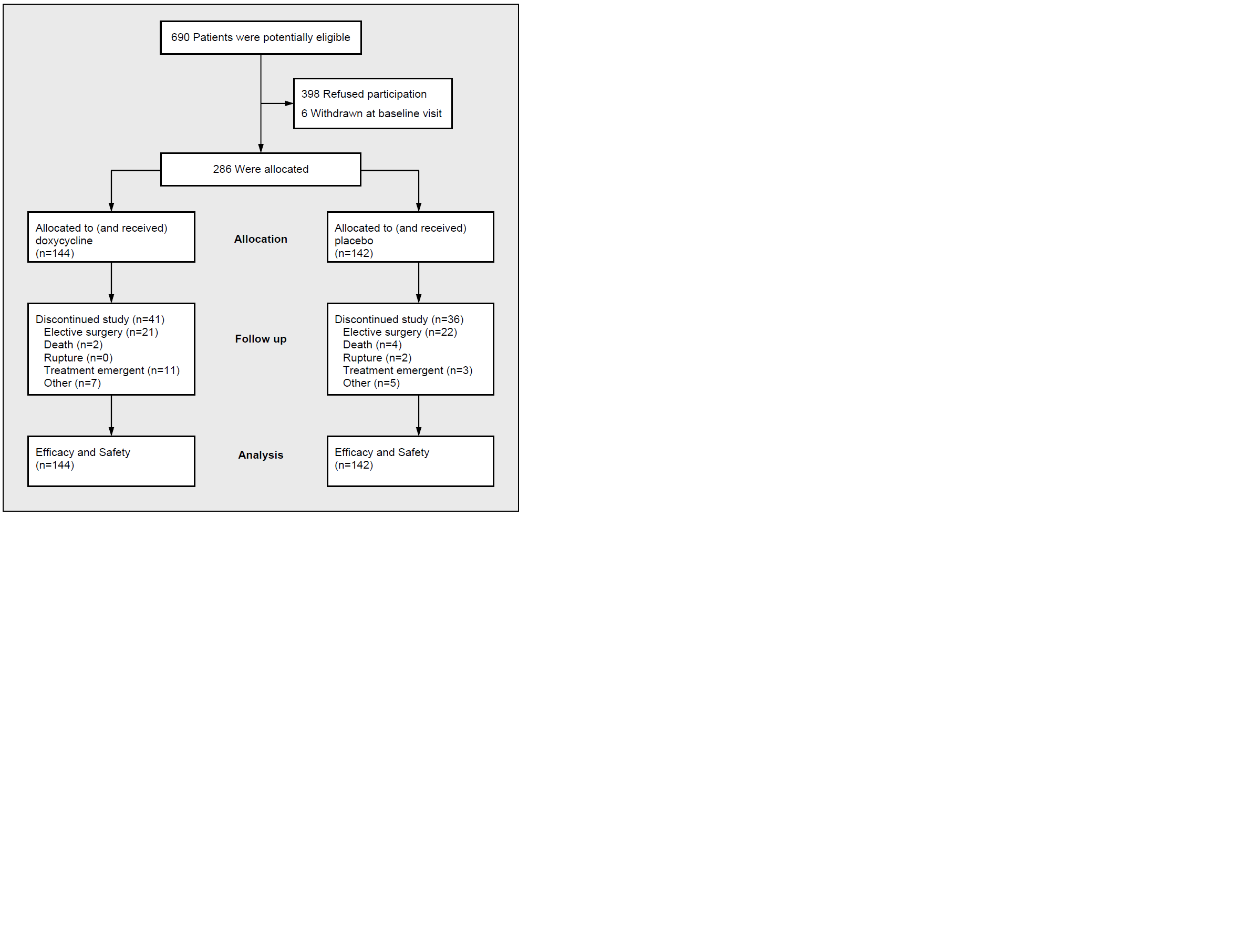

Supplement: Figure S1 — Consort diagram of the PHAST study [17]. (TIF) [file pone.0111952.s001.tif]
